# Managing the 1920s’ Chilean educational crisis: A historical view combined with machine learning

**DOI:** 10.1371/journal.pone.0197429

**Published:** 2018-05-30

**Authors:** Francisca Rengifo, Gonzalo A. Ruz, Aldo Mascareño

**Affiliations:** 1 School of Government, Universidad Adolfo Ibáñez, Santiago, Chile; 2 Faculty of Engineering and Sciences, Universidad Adolfo Ibáñez, Santiago, Chile; 3 Research Center Millennium Nucleus Models of Crises (NS130017), Santiago, Chile; The University of Sydney, AUSTRALIA

## Abstract

In the first decades of the 20^th^ century, political actors diagnosed the incubation of a crisis in the Chilean schooling process. Low rates of enrollment, literacy, and attendance, inefficiency in the use of resources, poverty, and a reduced number of schools were the main factors explaining the crisis. As a response, the Law on Compulsory Primary Education, considering mandatory for children between 6 and 14 years old to attend any school for at least four years, was passed in 1920. Using data from Censuses of the Republic of Chile from 1920 and 1930, reports of the Ministry of Justice, the Ministry of Education, and the Statistical Yearbooks between 1895 and 1930, we apply machine learning techniques (clustering and decision trees) to assess the impact of this law on the Chilean schooling process between 1920 and 1930. We conclude that the law had a positive impact on the schooling indicators in this period. Even though it did not overcome the differences between urban and rural zones, it brought about a general improvement of the schooling process and a more efficient use of resources and infrastructure in both big urban centers and small-urban and rural zones, thereby managing the so-called *crisis of the Republic*.

## Introduction

Since the 19^th^ century, education has been a central topic of discussion in Chile and other Latin American countries [1–6]. Besides its cognitive function, public education was politically considered as a means to transfer democratic, republican values to young people, aiming to enhance a sense for national unity and citizenship [7]. A crisis in education was thus regularly regarded as a general societal crisis, or vice versa, political problems were frequently attributed to the education system [8].

Over the first decades of the 20^th^ century, particularly between 1910 and 1920, social and political actors diagnosed the incubation of a crisis in the Chilean schooling process. More than half of the children between 5 and 14 years old remained excluded from the primary school in 1920 [9].

The Chilean educational system was formally established in 1842. The Central Statistical Office, created in 1843, was in charge of developing the technical tools for both measuring the literacy of population and assessing the expansion of the schooling process. The national census was the official source of information allowing the educational authority to identify the demographic basis for the spatial distribution of schools. This census, first applied in 1854 and every ten years since then, classified the population by sex, age, marital status, occupation, and additionally recorded who could read and write. On the basis of the data obtained from these sources, the first national Primary Education Act of 1860 established the foundation of two separate schools (one for boys and one for girls) every two thousand inhabitants. The expectation behind the Act was that by 1900 the primary school system should have covered the entire population of children under 14 years old, thereby enhancing the basis of inhabitants able to read and write. This goal was far from being achieved by the end of the 19^th^ century, thus unleashing a massive debate on the institutional foundations of the country. Illiteracy of young people was, therefore, not only an educational problem but mainly a political one, and as such it was denounced as a *crisis of the Republic* in the early 20^th^ century [10–15].

As a response to the incubated crisis [16], the Law on Compulsory Primary Education (Law 3654) was passed in 1920 [17]. The parliamentary debate on the topic began in 1900 aiming at enhancing the role of the State in education. The relevance of the problem led to immediate measures. In the twenty years of discussion, the number of primary schools almost tripled from 1,248 public schools covering a number of 114,565 students in 1895 to 3,214 public schools with the enrollment ascending up to 346,386 students in 1920 [5]. The Law considered mandatory for children between 6 and 14 years old to attend any school for at least four years. Poverty was not an exception. Only if the school was more than 4 km away from home, the child was exempt. The analysis of the data shows that the Chilean schooling process expanded in the considered period: the average enrollment rate of school-age children was 17.9% in 1895; 25,1% in 1920; and 46,1% in 1930. Until 1920, however, annual attendance, as the percentage of children present at public schools from the total enrollment, showed a persistent stagnation (62.8% in 1895 and 61.4% in 1920) [9]. Although enrolled, children were not attending to school. This situation changes in the period 1920-1930 as the Law on Compulsory Primary Education came into force. The percentage of enrolled children effectively attending to school increases to 70.5% in 1930 [9].

From a socio-historical perspective, two arguments aim to explain the stagnation in school attendance until 1920. The first one strengthens the institutional limitations of State for school enrollment [6, 18–22]. Considering the significant growth in both the number of schools and enrollment over the first decades of the 20^th^ century, this argument is rather weak, for it does not consider the investment in school infrastructure throughout the country and the significant growth of enrollment. The second argument addresses sociological factors. It emphasizes the limitations of poor families, particularly in rural and semi-urban areas, for sending children to school, such as having no shoes, long walking distances, topographic obstacles, and the crucial contribution of child labor to the domestic economy among poor families [5, 23]. While this argument seems to be sensitive to the particular living conditions of poor families, it rather underrates the impact of the Law on Compulsory Primary Education (1920) on the general performance of the schooling process in Chile in the period 1920-1930.

By combining historical analysis with machine learning techniques applied to data collected from historical sources, this article assesses the impact of the institutional changes of the period 1920-1930 on the management of the so-called *crisis of the Republic*. The period begins with the promulgation of the 1920’s Law on Compulsory Primary Education and ends up with the 1929’s world economic affecting the national budget for primary education in 1930.

Regarding the data structure, we have used 23 attributes containing multidimensional socio-demographic information as well as education-related data for each one of the 83 political and administrative units (departments) of the country in 1920, which merged into 65 departments in 1930. We argue that the Law on Compulsory Primary Education had a general positive effect on the Chilean schooling process in the analyzed period. Although it does not overcome the differences in school performance between urban and rural zones, it does manage to increase attendance, professionalize teaching, and favor a more efficient use of educational infrastructure throughout the country, thereby contributing to manage the diagnosed *crisis of the Republic*.

Applying machine learning techniques to data from historical archives is a rather new interdisciplinary field (see next section), and it is certainly a novel approach to the history of education in Latin American countries. When combined with historical and sociological analyses, these techniques help in assessing the foundations of education systems in the region. In the next section, we describe the data as well as the quantitative techniques applied. The results are presented in the second section. Finally, we discuss our results and relate them with the historical and sociological context.

## Materials and methods

### Machine learning techniques

The capacity to extract knowledge from multidimensional data can be addressed through data mining, in particular, using machine learning techniques. There are *unsupervised learning* techniques, which try to discover the natural groupings of the data, i.e. clusters, in order to identify different behaviors within the entire dataset. Another approach is through *supervised learning* techniques, which are capable of constructing, using a training set with previously labeled data, a classifier to predict the class label of a new data point.

The use of these two approaches, unsupervised or supervised, allows identifying patterns and relations (linear and nonlinear) amongst the variables when analyzing data from historical archives. For example, in [24] supervised learning techniques were employed for record linkage procedures in the study of social mobility between Europe and North America using censuses thirty years apart (1850-1880). Also, record linkage, geographic keywords, and surname analysis was used in [25] to identify the Irish in the long eighteenth century London in the 1841 census. Unsupervised and supervised learning techniques have been also used for analyzing contemporary census data as in [26]. The authors identify demographic features of socially disadvantaged groups in Taiwan through population and household data collected in the 2000 census. The problem of predicting the level of household car ownership as a function of the features of the household and the individuals that make up the household using the 2001 United Kingdom Census was addressed employing several supervised learning techniques (classifiers) for prediction purposes [27]. The effects of two major shocks to the Australian economy were analyzed in [28] using k-means (unsupervised learning) and the census data from 2001, 2006, and 2011. The analysis of the Indian 2011 census data for policy implementation purposes was conducted in [29] through the use of decision trees.

The advantages or strengths in using a machine learning approach to analyze data from historical archives and censuses in general is the capacity to automatically identify patterns (behaviors through time) and relations among the variables in a multidimensional way. Depending on the number of variables, this would be difficult or even impossible for a researcher using a traditional approach like descriptive statistics or interpretative work, as history regularly does. Moreover, machine learning techniques require none or very little user intervention, thereby reducing the possibilities of introducing biases in the analysis of historical data. Also, in the case of supervised learning or predictive models, these techniques allow exploring future outcomes or scenarios given past or present information (data), which may help when assessing the effects on the population of past (or current) policies, laws, or incentives.

On the other hand, one of the main weaknesses of using a machine learning approach is that these techniques are data driven and, therefore, rely or assume that the data is correct and reliable. Hence, a preprocessing stage is fundamental to reduce any errors or misleading analyses. Traditionally, machine learning algorithms work on one input data matrix constructed from several sources (that could even have different formats). So the first challenge is to merge our different sources of information into one input data matrix. The input data matrix should be carefully inspected to identify missing values and typographical errors, which are common in data from historical archives that are manually transcribed into digital formats. Then, once the input data matrix has been validated, it is important to consider the type of machine learning algorithm that will be employed. This implies different types of preprocessing, like the discretization of continuous variables or, in the case of algorithms that use distance measures like the clustering algorithm used in this work, for which it is vital for the data to be standardized or normalized. Therefore, a good preprocessing stage can have a significant impact on the outcome of the models. For more details on data handling and preprocessing for machine learning the reader can refer to [30] and [31].

### Input datasets for clustering the schooling distribution

The analysis is grounded in two datasets, one with demographic variables and the other one is schooling. As previously stated, the datasets were elaborated using the Censuses of the Republic of Chile of 1920 and 1930, reports of the Ministry of Justice, Culture and Education, of the Ministry of Education, and the Statistical Yearbooks 1895-1930. The ten-year series that compose the censuses was the first systematic effort to identify the demographic basis for developing a public school offer. The demographic dataset considers sex, age cohorts, literacy, and the type of agglomeration urban/rural at the provincial and departmental levels.

**Fig 1:**
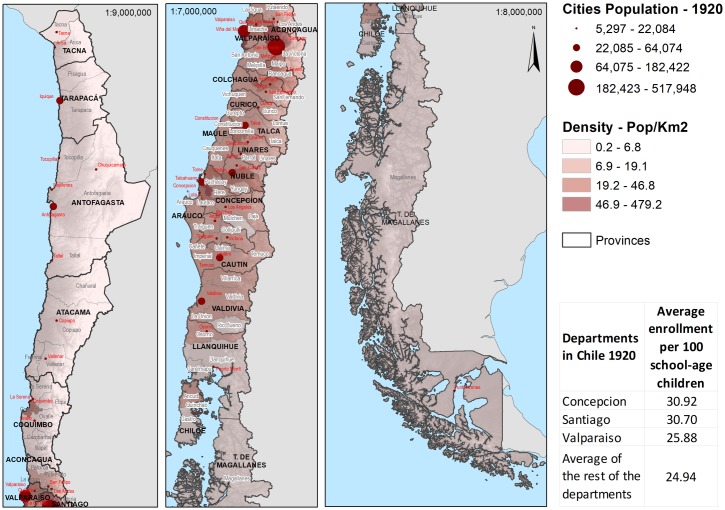
Map of Chile 1920.

**Fig 2:**
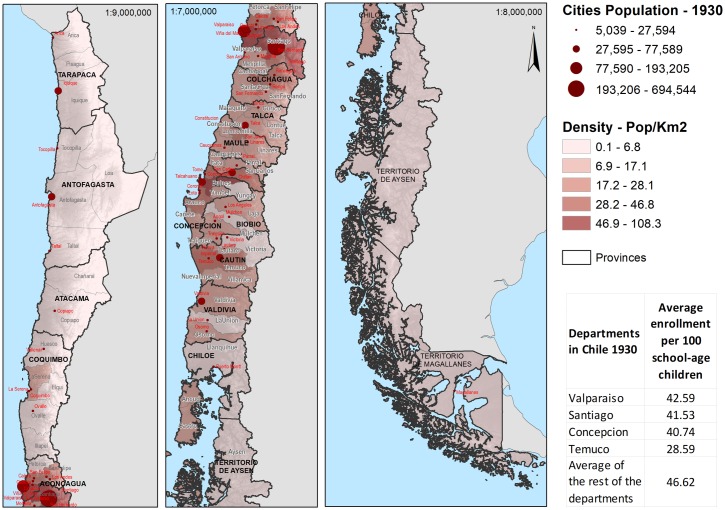
Map of Chile 1930.

These political units increased from 8 provinces in 1826 to 19 in 1883, and 25 in 1940. In turn, these were subdivided into departments (see Figs 1 and 2). Statistics of schools and students were registered by the Ministry of Justice, Culture and Public Instruction (created in 1837), which reports figures disaggregated by provinces and departments. Their records, therefore, allow quantifying the infrastructure of the Chilean public education system in number, capacity, and number of teachers by school. The data is correlated to the national territory to identify variables in each province and department. For this study, we considered the data at the level of departments. They represent a more detailed level of information than provinces.

As a result of cross-referencing the information between the two sources, the schooling dataset contains the number of public schools by sex and coverage (school enrollment), thereby allowing us to identify the relevance of each variable and thus know the different rates and patterns presented by the geographical administrative units (departments). In 1920, there were 83 departments. For each one of these, considering the two data sources, we filtered irrelevant and incomplete attributes, thus using the remaining 23 attributes containing socio-demographic and educational related information. The complete list is shown in Table 1.

**Table 1:** Attributes measured in 1920 and 1930 for clustering analysis.

Attribute name	Description
Urban_M	The number of males in an urban area
Urban_W	The number of women in an urban area
Rural_M	The number of males in a rural area
Rural_W	The number of women in a rural area
Single_M	The number of males that are single
Single_W	The number of women that are single
Married_M	The number of males that are married
Married_W	The number of women that are married
Widowed_M	The number of males that are widowed
Widowed_W	The number of women that are widowed
Read_M	The number of males that know how to read
Read_W	The number of women that know how to read
ReadNo_M	The number of males that do not know how to read
ReadNo_W	The number of women that do not know how to read
R6-14_M	The number of males between 6 and 14 years old that know how to read
R6-14_W	The number of women between 6 and 14 years old that know how to read
RN6-14_M	The number of males between 6 and 14 years old that do not know how to read
RN6-14_W	The number of women between 6 and 14 years old that do not know how to read
TotalSchools	The total number of schools
Norma	The number of normalist teachers
NonNorma	The number of non normalist teachers
TTeachers	The total number of teachers
Enrollment	The total number of children enrolled in schools

For 1930, the same 23 attributes were used for the existing 65 departments. The spatial location of the departments in 1920 is shown in Fig 1, whereas the spatial location of the departments for 1930 appears in Fig 2.

Several small departments from 1920 merged in the period 1920-1930, maintaining the name of one of them, or were included into a larger department. Nevertheless, the main departments of 1920 still remain in 1930. Therefore, this difference in the number of departments does not alter significantly the comparison between 1920 and 1930.

The maps in Figs 1 and 2 show the Chilean topography, the department’s territorial size and their population density. Also the major urban centers (more than 5000 inhabitants) are identified proportionally to the number of their city dwellers. Both maps show the large span of latitude of the Chilean national territory. The geographic common terminology identifies Big North (‘Norte Grande’), Small North (‘Norte Chico’), Central Valley, South, and Far South [32]. The same geophysical configuration is common to all zones: the huge Cordillera of the Andes to the east, and the Pacific Ocean to the west. The Big North: Tacna, Tarapacá, Antofagasta, conformed 24% of the total national territory; the major economic activity is mining. This region is next to the Small North (16% of the national territory), also a mining zone with an important agricultural production. The Central Valley is the core of the national territory. With two major cities, Santiago (capital city) and Valparaíso (main port), the Central Valley concentrates the Chilean population. Its territorial size is equivalent to 11.2% of the Chilean territory, and it concentrated 40% of the agricultural land, being also a copper mining zone. The South Region begins with the provinces of Concepción and Arauco; its main economic activity was cereal agriculture (it represented 7% of the total agricultural land), and livestock. Also an important wood industry and coal mining were developed in this region. The South region ends in the Lake District, whose central economic activity was the wheat production, livestock, and dairy. There is a maritime channels zone (Far South) that occupied 33% of the national territory and whose economic activities were logging and agriculture at a smaller scale. The Far South is a vast territory sparsely inhabited. Finally, the legend in the maps shows the average enrollment per 100 school-age children for the big cities and the average for the rest of the departments.

A description of the datasets used in this work appears in Table 2. As said, for 1920, there are 83 instances each one representing a department. Each department is characterized by the 23 attributes of Table 1. For 1930, there are 65 instances, each one characterized by the same 23 attributes. A general comparison is given between educational indicators computed with the educational-related attributes. A t-test with a level of significance of *alpha* = 0.05 was performed to see whether the differences between 1920 and 1930 are statistically significant for these indicators. Table 2 presents the *p*-values and the effect sizes computed by Cohen’s *d* measure. From these values we can conclude that the difference (increase) shown from 1920 to 1930 is statistically significant and meaningful for the four indicators, being the difference slightly lower in the average number of schools per 100 school-age children.

**Table 2:** Description of the datasets and educational indicators computed using the attributes.

Datasets and statistics	Number of instances	Number of attributes	Average literacy rate	Average num. schools per 100 school-age children	Average num. teachers per 100 school-age children	Average num. enrollment per 100 school-age children
1920	83	23	0.452	0.221	0.481	25.104
1930	65	23	0.708	0.362	0.889	46.115
*p*–value	-	-	2.2e-16	1.018e-08	2.2e-16	2.2e-16
Cohen’s *d*	-	-	2.465	1.089	1.992	2.030

It is important to point out that for the clustering and decision tree analyses presented later on, the order in which the 23 attributes (variables) appear in the datasets does not affect the results. Both of these methods are not sensitive to the order of the attributes. Therefore, the order in which the attributes appear in Table 1 does not represent relevance.

### *k*-means clustering

In this work we are interested in finding flat (one-level) clusters, with no hierarchical structures. Among the partitional clustering techniques, we propose to use *k*-means due to its simplicity, effectiveness, and the existence of a global cost function which can be used to monitor convergence. Also the data type is numeric, therefore the Euclidean distance can be used as a similarity measure in the algorithm. An alternative to partitional clustering would be the use of an agglomerative hierarchical clustering approach, such as Ward’s clustering, which is in fact *k*-means correct hierarchical analog [30], thus yielding similar results.

The *k*-means clustering algorithm [33] is a method for finding *k* vectors *c*_*j*_ (*j* = 1, 2, …, *k*) that represent an entire dataset. The data is partitioned into *k* clusters, with each cluster represented by its mean vector and each data instance *x*_*i*_ assigned to the cluster with the closest vector. At each stage, the *N* data examples *D* = {*x*_1_, …, *x*_*N*_} are partitioned into *k* disjoint clusters *D*_*j*_, each containing *N*_*j*_ instances. A cost function of dissimilarity (or distance) known as the sum of the squared error (SSE) is defined as

$$\begin{array}{c} J=\sum_{j=1}^{k}J_{j}=\sum_{j=1}^{k}\sum_{x_{i}\in D_{j}}{∥x_{i}-c_{j}∥}^{2}, \end{array}$$ (1)

where *c*_*j*_ is the center of the *j*th cluster given by the mean of the data instances belonging to that cluster; namely:

$$\begin{array}{c} c_{j}=\frac{1}{N_{j}}\sum_{x_{i}\in D_{j}}x_{i}. \end{array}$$ (2)

The algorithm works by first initializing a random partition of the dataset, then an iterative process is carried out consisting of two steps until no further changes to (1). These steps are:

1. The mean vectors *c*_*j*_ for each cluster are calculated using (2).
2. Rearrange the clusters: each data instance *x*_*i*_ is assigned to the *j*th new cluster if *x*_*i*_ is closer to *c*_*j*_ than to the other mean vectors.

Nevertheless, one of its drawback (shared by other clustering techniques) is that the user must define beforehand the number of clusters *k* to be found. An exception to this problem is when, for example, hierarchical clustering is used which does not require the user to give beforehand the number of clusters to run the algorithm. Although once the data is organized into hierarchical groups (clusters) and visualized through a dendrogram (tree representation), the user must specify where to cut the dendrogram, either directly by saying how many clusters are required or by specifying a threshold value on a measure computed from the dendrogram like the inconsistency coefficient.

The specification of the number of clusters presents a difficulty if there is little or no knowledge about the natural grouping of the dataset. To overcome this problem we will use the following strategy. First, we will make a linear projection of the 23-dimensional dataset to a 2-dimensional plane using principal component analysis (PCA) [34]. This will allow us to identify outliers that can be considered as clusters by themselves (thus removing them from the dataset when *k*-means is run) as well as to identify visually a plausible value for *k*. The second approach is to plot the SSE versus the number of clusters. The idea here is to find where there is an elbow or dip in the plot indicating the natural clusters. With this method, the exact value where the elbow occurs is not always clear; for this we will complement the result with the next method. The third approach is to use a cluster validity index, in particular, the Dunn index [35], to identify an appropriate value (or values) for *k*. We use this index to find compact clusters with a small amount of variance within each cluster yet as separated as possible. In order to compute the index, one needs the dataset and the resulting clustering labels obtained by applying any clustering technique. In this case, we will use *k*-means. Different values of the Dunn index are obtained for different values of *k*. A higher Dunn index indicates better clustering.

We would like to emphasize that although we use in this work a partitional clustering approach to look for flat clusters, in particular *k*-means, a hierarchical clustering approach could have been used as well (even though we are not searching for hierarchical structures within the data), in particular, Ward’s method or Average linkage method, which share similarities with *k*-means, therefore, yielding similar clustering results.

Once we have identified the clusters for 1920 and 1930, we identify the most relevant attributes and rules that are capable of explaining why a department belongs to a specific cluster. For this we will use a decision tree algorithm *rpart* [36] that automatically selects the most relevant attributes (with higher discriminatory power) capable of classifying a department into a specific cluster through explicit rules based on splitting conditions using the attributes, carried out using the Gini index. It is important to point out that the datasets are standardized (mean = 0 and standard deviation = 1) before applying any of the above-described techniques. To evaluate the predictive performance of the resulting trees, we will perform *k*-fold cross validation [31]. This procedure consists in randomly partitioning the original dataset in *k* equally sized (more or less) groups. Then, *k* − 1 partitions are used to train the decision tree, and the remaining partition is used for testing. This process is repeated *k* times, so that each partition is used as a test set once. We use *k* = 10, as recommended in [31]. Within each fold we will compute the *accuracy*, which is the fraction of correct classifications, and the *kappa* statistic, which compares the accuracy of the trained model with the accuracy of a random model. While the maximum value for both measures is 1 (optimal classifier), for this application, the kappa statistic is more appropriate given the multi-class classification problem at hand. To interpret its value, a characterization proposed in [37] is commonly used: values ≤0 as indicating poor agreement, 0 − 0.2 as slight, 0.21 − 0.4 as fair, 0.41 − 0.6 as moderate, 0.61 − 0.8 as substantial, and 0.81 − 1 as almost perfect agreement.

At the end of the *k*-fold cross validation process, we compute the mean and the standard deviation of the accuracy and the kappa statistic to obtain a final estimation of the predictive performance of the decision trees. Once the evaluation process is over, we will use all the dataset to train a decision tree for each year (1920 and 1930).

## Results

### PCA results

**Fig 3:**
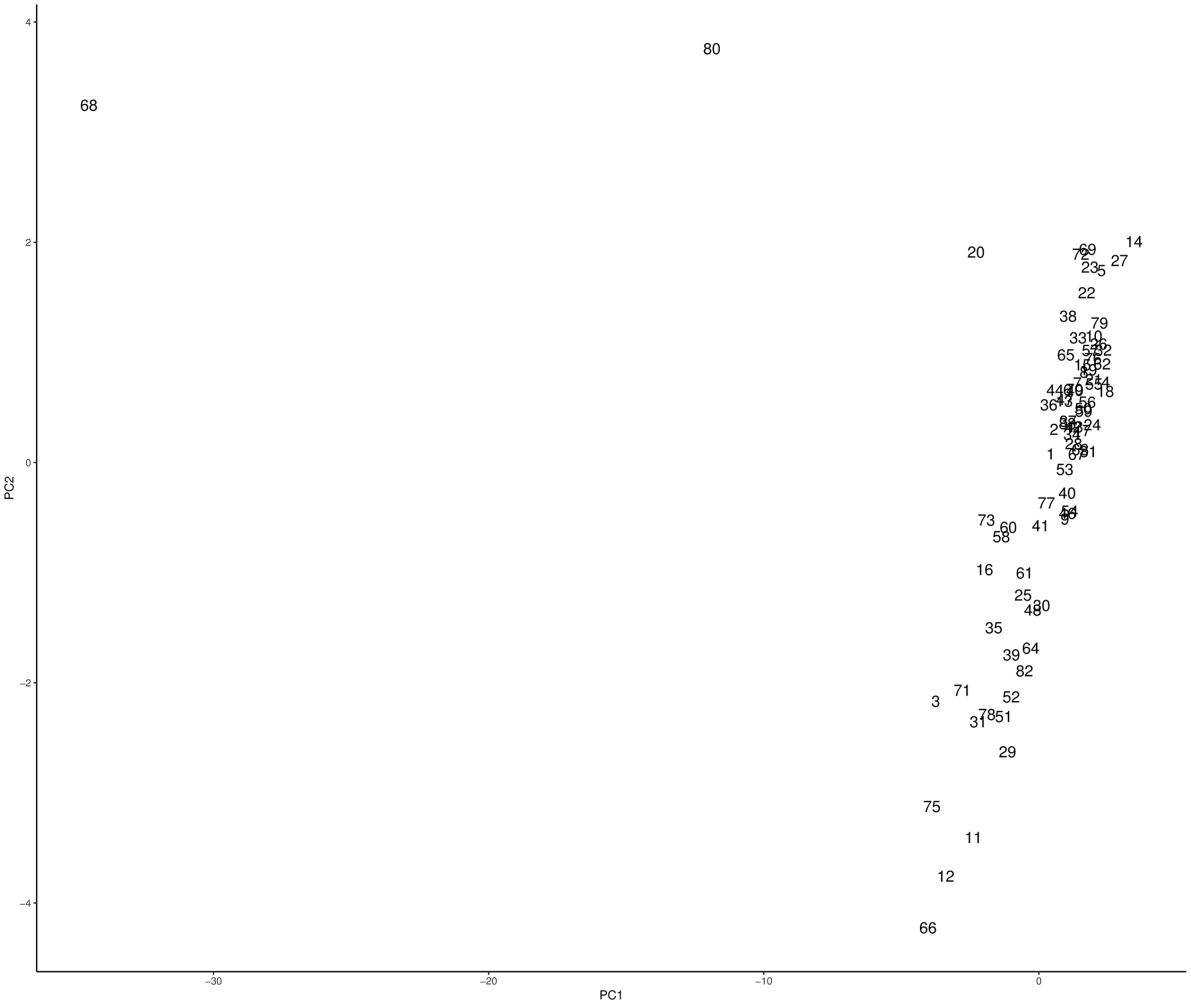
PCA projection of the 1920 dataset. Number labels are associated to each department.

**Fig 4:**
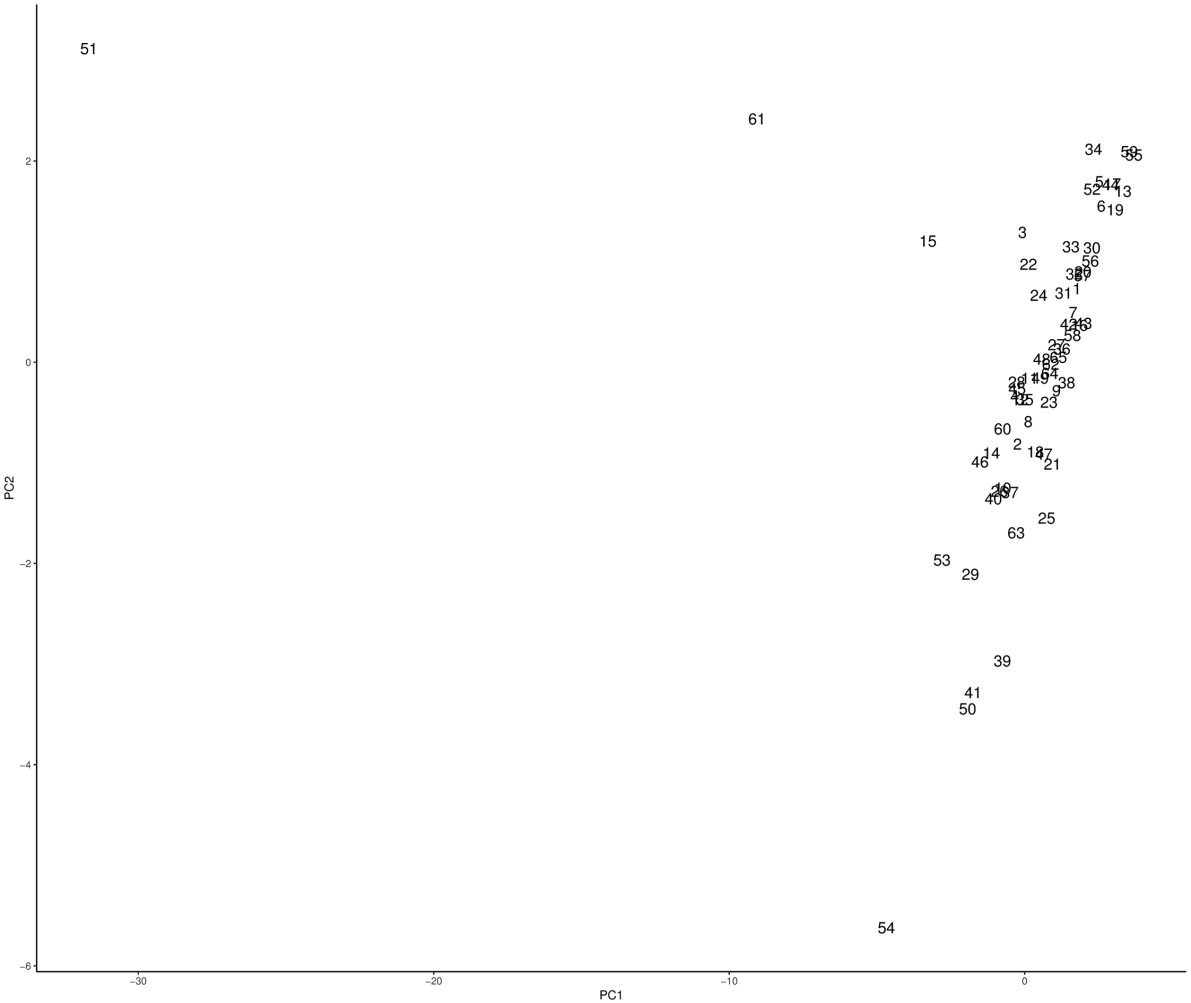
PCA projection of the 1930 dataset. Number labels are associated to each department.

Figs 3 and 4 show the resulting PCA projections for 1920 and 1930 respectively. The number labels on each data point are used to identify each department. In Fig 3 we can identify three outliers, Santiago (68), Valparaíso (80), and Concepción (20), two of them in the Central Valley (Santiago and Valparaíso) and one in the southern region. Also, from the cloud of points on the right, we can visually identify 2 or 3 clusters. Before analyzing Fig 4, it is important to point out that given that in 1930 the number of departments changes (from 83 in 1920 to 65 in 1930), the labels are not the same as for 1920. We notice that in Fig 4, Santiago (51), Valparaíso (61), and Concepción (15) are still outliers, although Concepción appears closer to the big cloud of points to the right. Nevertheless, as we will discuss in the next subsection, Concepción still behaves as an outlier (unique cluster). Also, we notice a new outlier, this is Temuco (54) (South). For this dataset, apart from visually identify two clusters, one could explore more than three.

### SSE and cluster validity index plots results

**Fig 5:**
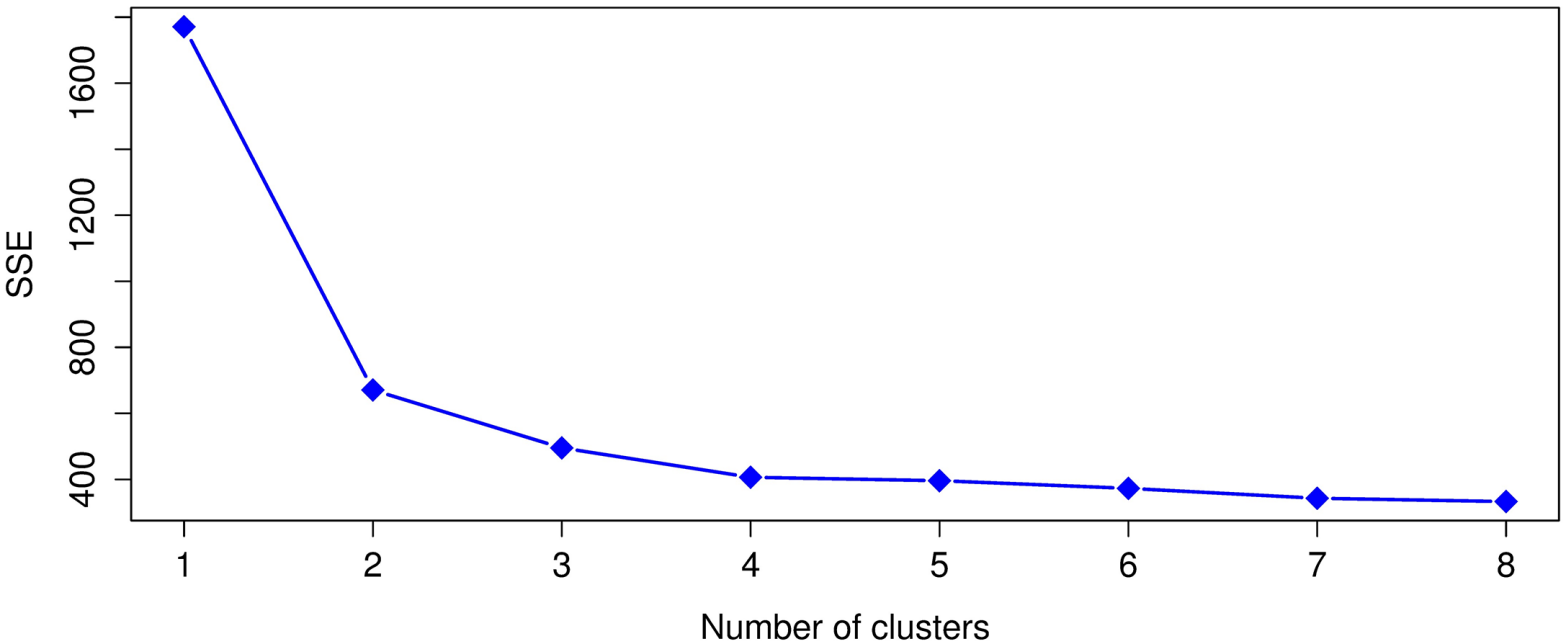
The sum of squared errors (SSE) for the 1920 dataset.

**Fig 6:**
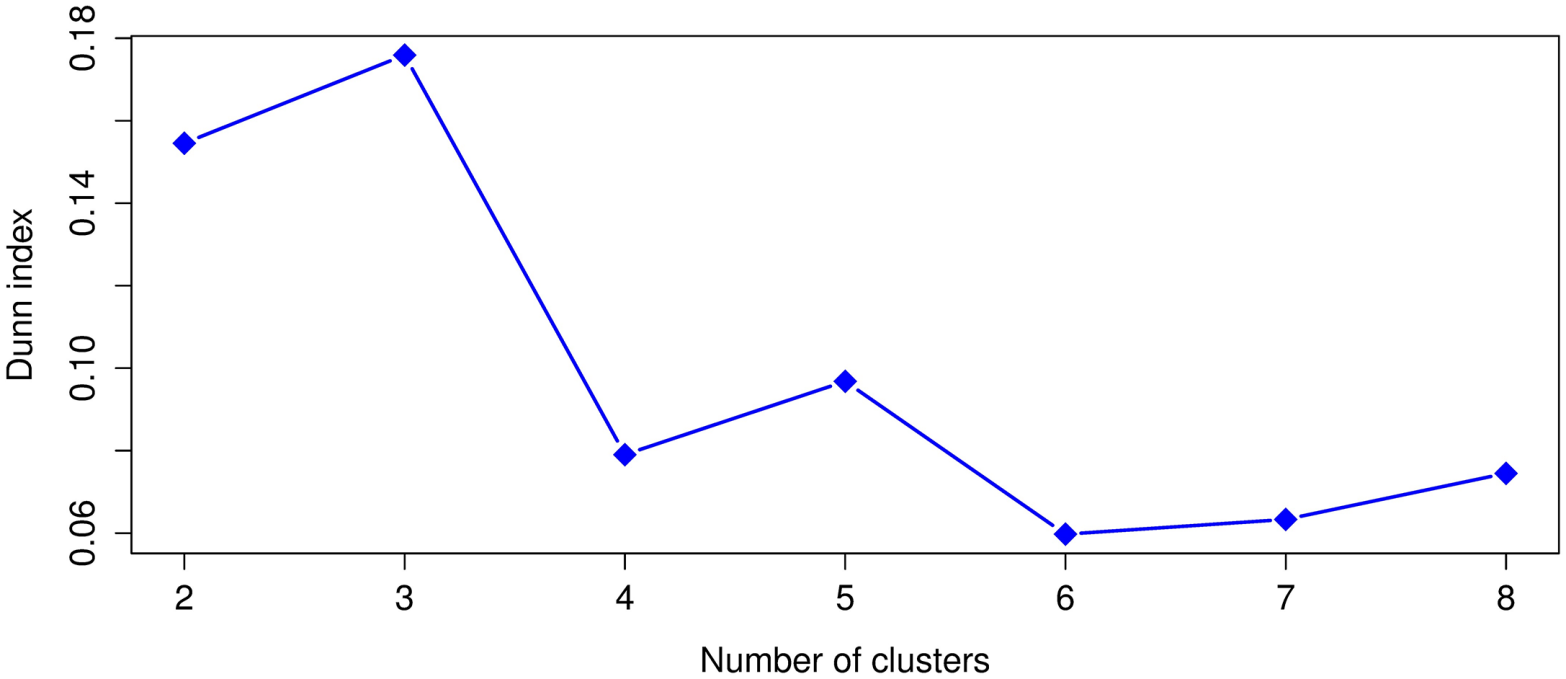
Dunn index values for the 1920 dataset. A higher Dunn index indicates better clustering.

**Fig 7:**
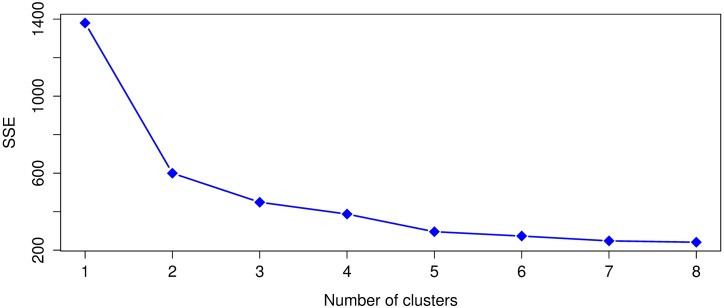
The sum of squared errors (SSE) for the 1930 dataset.

**Fig 8:**
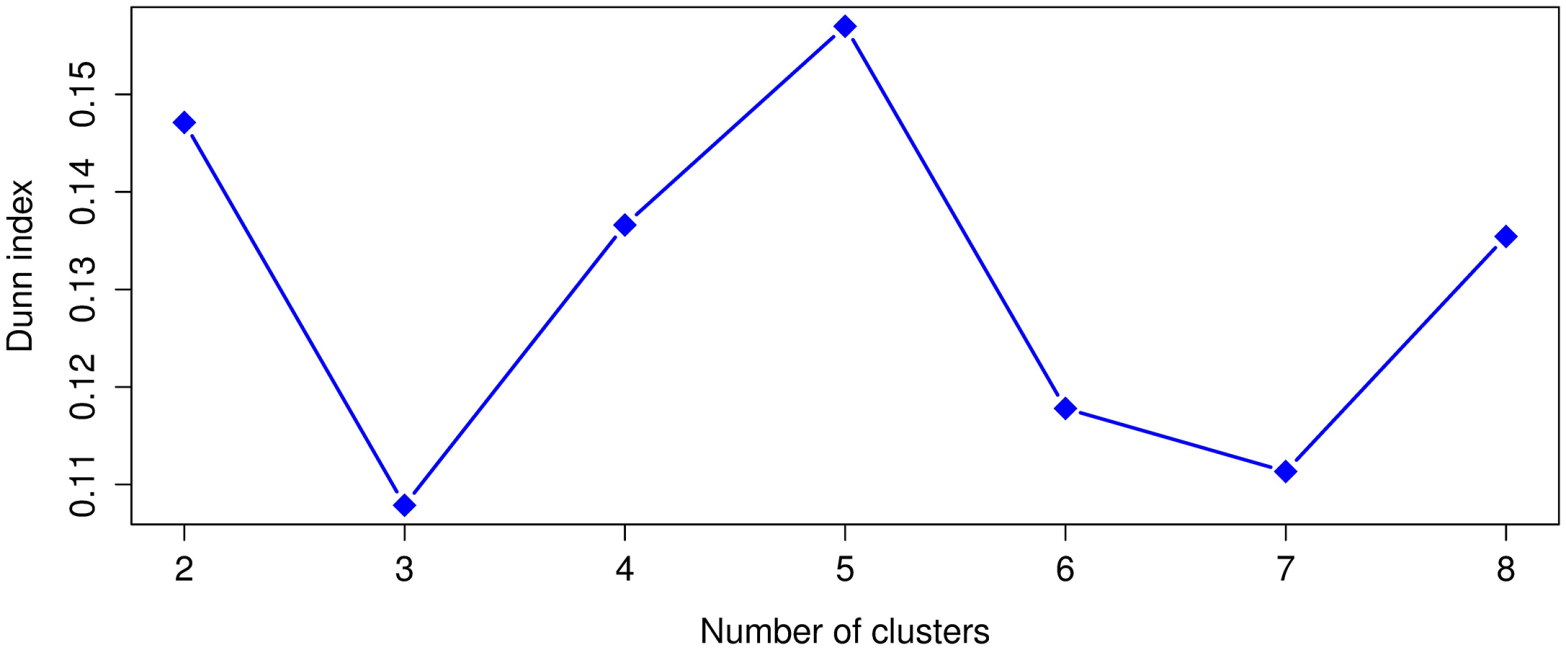
Dunn index values for the 1930 dataset. A higher Dunn index indicates better clustering.

Leaving out the outliers identified in the previous subsection, the SSE values from (1) were computed for *k* = 1 to *k* = 8 and the Dunn index for *k* = 2 to *k* = 8. Given that *k*-means uses a random initialization that can affect the convergence of the algorithm, we ran 30 times *k*-means for each *k* and selected the clustering result, for each *k*, with the lowest value of SSE (1). The SSE and Dunn index results for the 1920 dataset appear in Figs 5 and 6. Whereas the SSE and Dunn index results for 1930 are respectively shown in Figs 7 and 8.

For 1920, we notice with the SSE values that *k* = 2, 3, 4 are in the elbow region of the plot. In the case of the Dunn index the best clustering results occurs with *k* = 3 and in the second place with *k* = 2. For 1930, in the case of SSE, the elbow region is for *k* = 2, 3, 4, 5 and for the Dunn index the best clustering occurs with *k* = 5 and the second best with *k* = 2. We see for both years that the best or more plausible cluster number obtained by the Dunn index also lies within the elbow region of the SSE plots, therefore, we will use *k* = 3 for 1920 and *k* = 5 for 1930 in our analysis.

For 1930, we also considered to include Concepción given that it was closer to the other data points in this year. For 1930, we obtained six clusters as the most plausible ones, but when we analyzed the clusters one of them contained only one point and it was Concepción. Therefore, it is correct to still consider this department as an outlier.

### *k*-means results

**Fig 9:**
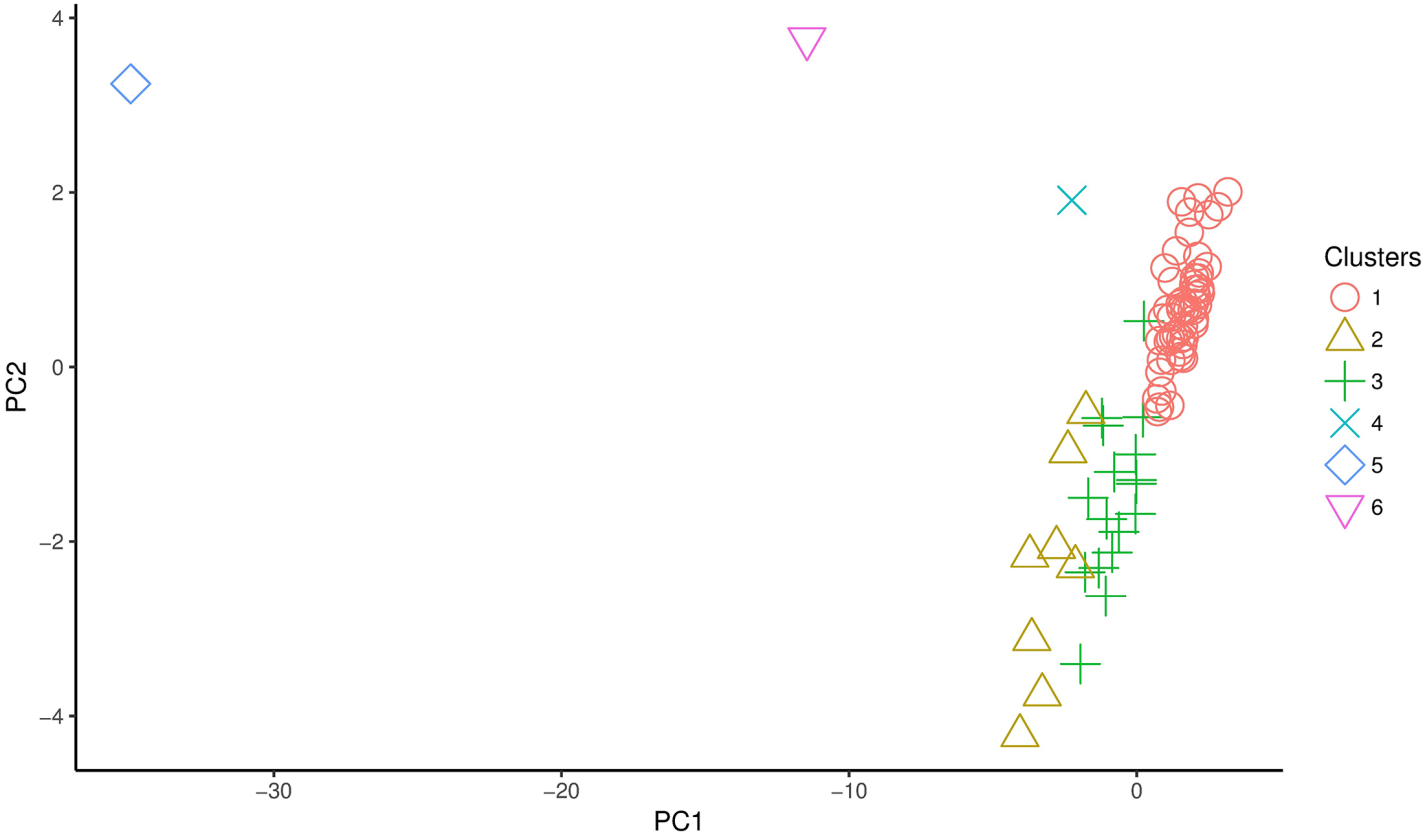
PCA projection of the 1920 dataset cluster labels.

**Fig 10:**
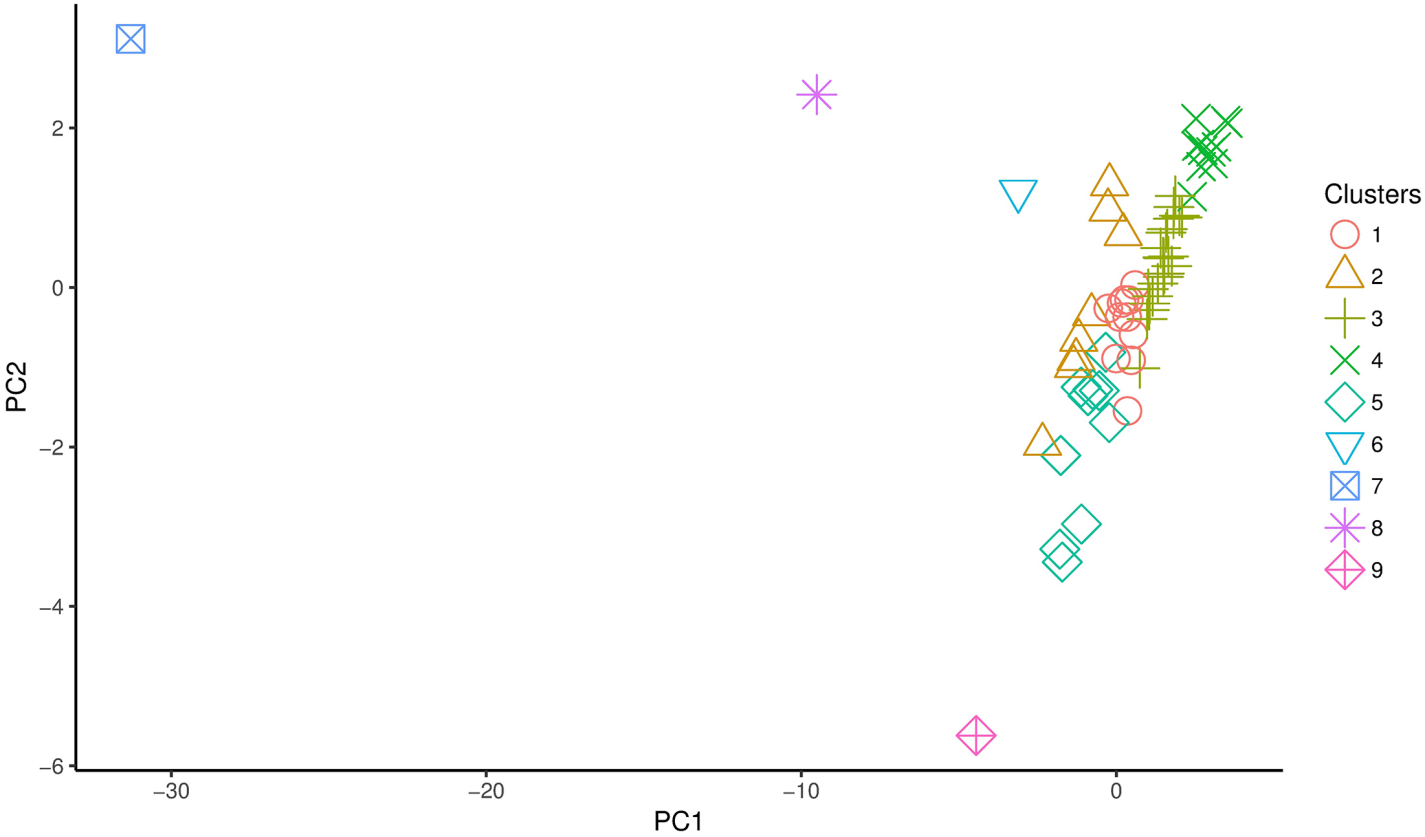
PCA projection of the 1930 dataset with cluster labels.

Using the clustering results from the previous stage with *k* = 3 for 1920 and *k* = 5 for 1930, and incorporating the outliers as unique clusters, Tables 3 and 4 present the clustering results with the 1920 dataset and the centroid of each cluster respectively, whereas Tables 5 and 6 show the results for the 1930 dataset and the centroid for each cluster respectively. Also Figs 9 and 10 show the PCA projection with the cluster labels.

**Table 3:** Clustering results with the 1920 dataset.

Cluster label	Departments
1	Ancud; Angol; Arauco; Arica; Bulnes; Cachapoal; Canete; Carelmapu; Casablanca; Cauquenes; Chanaral; Chanco; Coelemu; Collipulli; Combarbala; Constitucion; Copiapo; Coquimbo; Curepto; Elqui; Freirina; Illapel; La Ligua; La Serena; La Union; Lebu; Limache; Llaima; Loncomilla; Lontue; Los Andes; Maipo; Mariluan; Mulchen; Nacimiento; Parral; Petorca; Pisagua; Puchacay; Putaendo; Quinchao; Rio Bueno; San Antonio; San Felipe; Santa Cruz; Tacna; Tal Tal; Talcahuano; Tocopilla; Traiguen; Vallenar; Vichuquen; Yungay.
2	Antofagasta; Caupolican; Chillan; Magallanes; San Fernando; Talca; Tarapaca; Tarata; Temuco; Valdivia.
3	Castro; Curico; Imperial; Itata; La Laja; La Victoria; Lautaro; Linares; Llanquihue; Melipilla; Osorno; Ovalle; Quillota; Rancagua; Rere; San Carlos; Villarrica.
4	Concepcion.
5	Santiago.
6	Valparaiso.

**Table 4:** Centroid for each cluster with the 1920 dataset.

Attribute	C1	C2	C3	C4	C5	C6
Urban_M	3229	15218	7215	31665	229387	106890
Urban_W	3742	16056	8253	39465	281303	114055
Rural_M	8480	22460	20099	6147	23486	1770
Rural_W	7862	18804	18660	5385	19322	2454
Single_M	8449	26990	19621	26540	169607	73251
Single_W	8025	23035	18325	29803	188100	71220
Married_M	2875	10029	6865	10048	75419	31678
Married_W	2833	9855	6893	10430	85428	32826
Widowed_M	386.2	1123	832.5	1224	7847	3731
Widowed_W	746.6	2454	1697	4617	27099	12463
Read_M	10594	39552	23720	49594	338224	157864
Read_W	10067	33834	21811	55728	407158	162855
ReadNo_M	12745	36692	30633	25030	167202	59456
ReadNo_W	13405	36553	31723	33972	194292	70171
R6-14_M	2447	7609	5554	11050	68929	30542
R6-14_W	2407	7718	5256	11050	71910	30884
RN6-14_M	3230	8744	7728	5587	36381	13738
RN6-14_W	3036	8021	7367	6126	35588	14116
TotalSchools	25.83	58.78	51.29	45	245	92
Norma	20.17	61	35.35	200	1175	279
NonNorma	34.3	85.67	71.94	33	571	211
TTeachers	54.47	146.7	107.3	233	1746	490
Enrollment	2835	8927	5798	10456	65349	23102

**Table 5:** Clustering results with the 1930 dataset.

Cluster label	Departments
1	Cachapoal; Caupolican; Cauquenes; Curico; La Union; Linares; Maipo; Quillota; San Carlos; San Felipe; San Fernando.
2	Antofagasta; Arauco; Chillan; Iquique; La Serena; Rancagua; Talca; Valdivia.
3	Ancud; Bulnes; Canete; Constitucion; Huasco; Illapel; Itata; Lautaro; Loncomilla; Lontue; Los Andes; Mataquito; Mulchen; Parral; Petorca; Tocopilla; Tome; Traiguen; Victoria; Yumbel; Yungay.
4	Arica; Aysen; Chanaral; Copiapo; Elqui; Loa; Magallanes; Pisagua; Tal Tal; Tierra del Fuego; Ultima Esperanza.
5	Angol; Castro; Laja; Llanquihue; Melipilla; Nueva Imperial; Osorno; Ovalle; Santa Cruz; Villarrica.
6	Concepcion.
7	Santiago.
8	Valparaiso.
9	Temuco.

**Table 6:** Centroid for each cluster with the 1930 dataset.

Attribute	C1	C2	C3	C4	C5	C6	C7	C8	C9
Urban_M	14955	44270	7325	7282	14284	106634	667796	253896	40216
Urban_W	17713	48718	8994	6851	16990	131308	800978	279704	48428
Rural_M	36758	42249	26579	10039	60420	27686	113910	29732	88282
Rural_W	33322	34625	24920	6844	58734	22840	94186	24388	81640
Single_M	18045	29893	12021	6071	26805	46574	254718	93460	46417
Single_W	16534	26457	11198	4283	25656	49263	268763	90439	43289
Married_M	7167	12057	4418	2329	9649	18740	126400	43836	16191
Married_W	7374	12156	4509	2134	10037	20724	136686	46701	17285
Widowed_M	836	1326	510.6	260.6	998.5	1846	10915	4518	1641
Widowed_W	1790	3059	1105	430	2170	7087	42083	14906	4460
Read_M	4778	8419	3121	1511.5	7331	16288	79833	31283	12453
Read_W	4857	8492	3053	1352.2	7143	17541	88139	33276	11028
ReadNo_M	13012	25874	8279	5775	19348	45799	261414	99399	31034
ReadNo_W	12454	23577	7669	3961	17629	49467	301498	104166	26211
R6-14_M	2134	2110	1296	229.7	2780	2185	8050	3443	6687
R6-14_W	1817	1991	1226	228.5	2702	2471	8676	3321	7806
RN6-14_M	6667	7524	3929	875.3	7648	6950	27888	12956	16736
RN6-14_W	7019	8350	4638	912.5	9943	12550	40927	17662	22628
TotalSchools	51.36	53.12	36.1	10.91	74.8	76	290	135	60
Norma	56.91	124.6	40.48	17.45	79.2	313	1601	510	110
NonNorma	67.27	63.12	37.48	12.91	71.7	49	407	198	81
TTeachers	124.2	187.8	77.95	30.36	150.9	362	2008	708	191
Enrollment	6454	9096	4143	1630	7862	15682	76720	30381	10857

### Decision tree results

In order to identify key education-related attributes that can explain the clusters identified in the previous step, we apply the decision tree algorithm *rpart* using only the 13 education-related attributes in the datasets. We first evaluated the predictive performance of the decision trees trained with these educational attributes. For this we performed 10-fold cross validation. In the case of 1920, we obtained a mean (± standard deviation) accuracy of 0.87 ± 0.09 and a mean (± standard deviation) kappa value of 0.76 ± 0.14. For 1930, the predictive performance decreased, obtaining a mean (± standard deviation) accuracy of 0.75 ± 0.18 and a mean (± standard deviation) kappa value of 0.66 ± 0.21.

**Fig 11:**
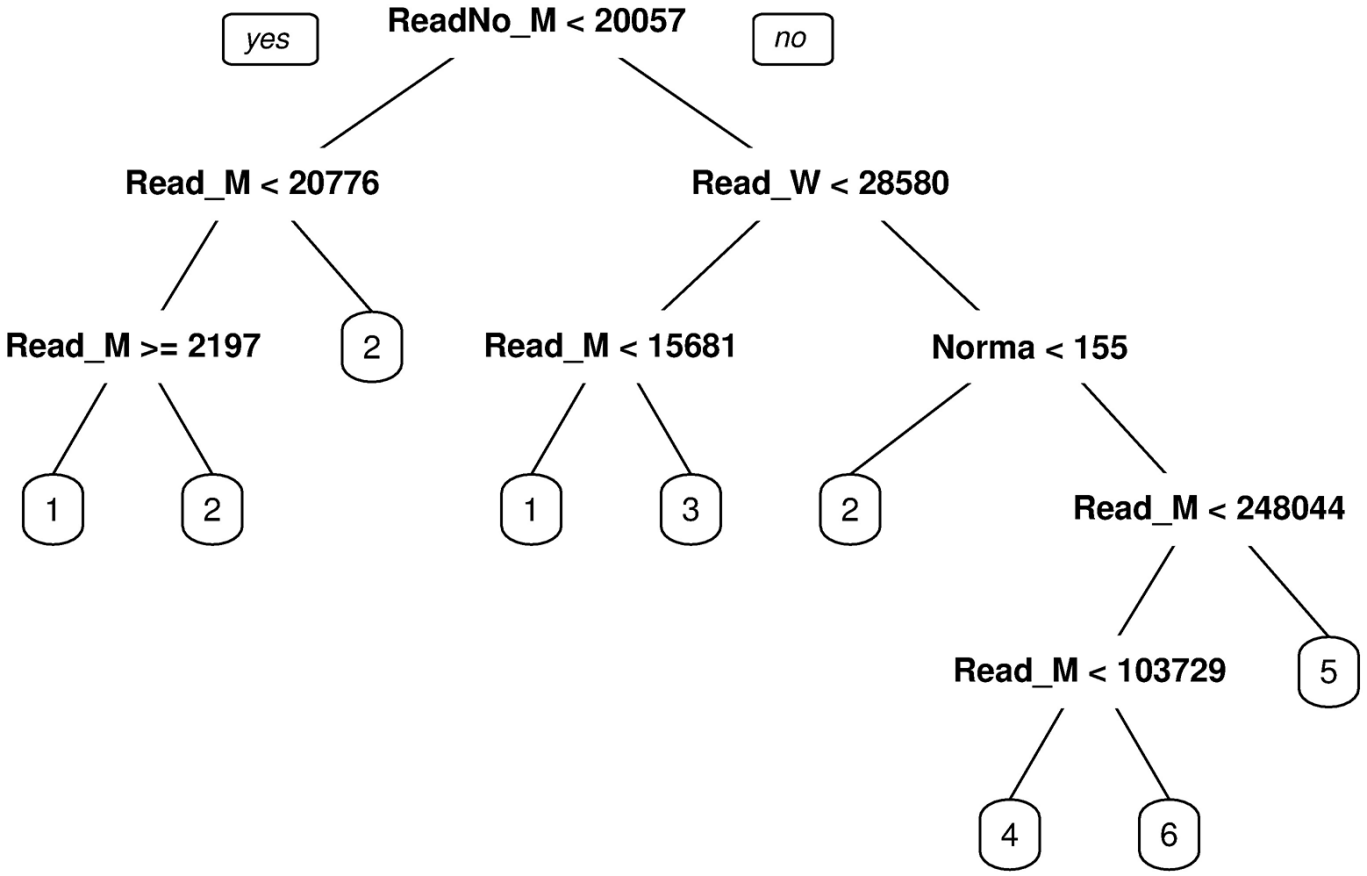
Decision tree explaining the cluster formations for 1920. The leaves of the tree contain the label of the clusters.

**Fig 12:**
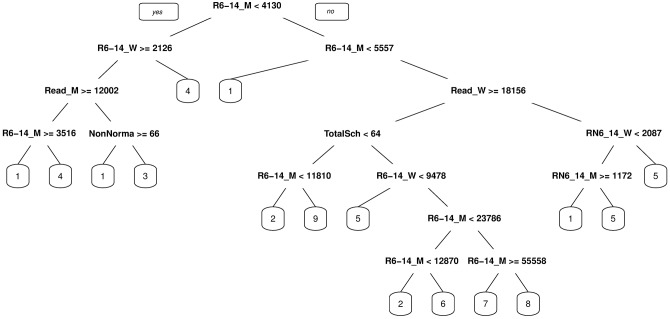
Decision tree explaining the cluster formations for 1930. The leaves of the tree contain the label of the clusters.

After the evaluation, we learned a decision tree using all the examples as a training set in each respective period. The resulting decision tree for 1920 appears in Fig 11 and for 1930 in Fig 12.

Each node of the tree contains a splitting condition using an attribute that the algorithm selects. If the condition is satisfied, then you follow left, else you continue right. For each instance (data point), you follow the path in the tree according to the conditions until you reach a final node (leaves of the tree) which contains the cluster label indicating to what cluster the instance belongs to.

## Discussion

The turn from the 19^th^ to the 20^th^ century signalized the end of the agrarian, elitist social structure in the Chilean society and the beginnings of a modern, mass society. As a consequence, strong pressures upon the old social institutions led to profound changes and reforms. This period (1900-1920) was known as *the crisis of the Republic*. Since public education was conceived of as the main instrument for social and political inclusion, it concentrated the discussions as to how to overcome the crisis. The 1920’s Law on Compulsory Primary Education and its deployment through different policies was a result of these discussions. The institutional reforms associated with this Law could not eliminate the gap in the schooling process between big city centers and the small-urban and rural zones. However, from the above-presented results there are several aspects to analyze that show the positive effects of the Law for both the large urban departments and the smaller ones, either urban or rural.

The results by using PCA show that the departments of Santiago, Valparaíso (Central region), and Concepción (Southern region) differ significantly from the rest of the departments and maintain that peculiarity between 1920 and 1930. In 1920, Santiago (capital city) and Valparaíso (the most important Chilean port) were the largest urban departments, and Concepción (the main port of the south) was the third largest source of industrial development at the national level. The three departments were densely populated trade centers and corresponded to the cities which by 1920 had grown faster than the national population rate [38]. These cities concentrated 23% of the total population in a predominantly rural country [9].

In 1930, the department of Temuco (Southern region) appears as a new outlier. Located on the northern edge of *Araucanía*, southern territory of Chile, Temuco led the expansion of the educational system in this region. State policies of internal relocation caused a growing displacement of inhabitants to urban centers, mainly to the city of Temuco [39]. In each inter-census period between 1895, 1907 and 1920, the city doubled its population because of the job offer provided by the forestry industry and the centralization of administrative and urban services. This led to a growing schooling demand [40].

In 1920, the average enrollment rate of school-age children in the three main clusters (Santiago, Valparaíso, and Concepción) was 29.17% while the average enrollment for the rest of the clusters amounted to 24.95% (smaller departments). This low enrollment rate was, therefore, not just an issue for small-urban and rural zones, but also for big cities, thus, justifying (amongst other educational indicators) the passing of the Law on Compulsory Primary Education in 1920. This Law aimed at increasing the enrollment and the capacity of the public school system to accommodate a greater number of students. As a consequence, the average enrollment rate of school-age children increased significantly in big cities to 38.37% in 1930 (*p*-value = 0.006, *d* = 1.71), and even more for small-urban and rural zones to 46.62% (*p*-value < 2.2e-16, *d* = 2.07), showing a general positive impact of the Law. Although it should be noted that while the enrollment rate of school-age children increased, the number of school-age children in small-urban and rural zones dropped from 1,350,637 in 1920, to 736,284 in 1930. However, this pattern is much less a result of the education policies than a consequence of the outcomes of a general urbanization trend whose roots were in the exhaustion of the agro-exporting model in Chile and Latin America at that time, being the 1929’s world crisis the tipping point of these developments [41].

A complementary way of visualizing the dynamics of the schooling process is through the SSE and Dunn index. When we consider the 23 socio-demographic and educational attributes, and excluding the three outliers in 1920, we found that the departments could be grouped in three clusters. Moreover, when we see the *k*-means clustering results, we noticed that in 1920, there is one predominant cluster (cluster 1) that contains 53 out of the 83 departments. This cluster includes departments from the north, central, and south of Chile, thus indicating a general homogenous behavior throughout the Chilean territory (excluding, of course, the urban outliers Santiago, Concepción and Valparaíso) (see Table 4). When we consider the 53 departments in cluster 1, we observe mainly rural zones. Cluster 2 is composed by bigger departments, being one of them Temuco, an outlier in 1930. And cluster 3 contains departments from Southern Chile mainly with indigenous population.

For 1930, also excluding the four outliers (Concepción, Valparaíso, Santiago, and Temuco), we identified five clusters, none of them being significantly predominant in size. Cluster 1 is composed by small cities; cluster 2 includes bigger cities (future regional capitals by the mid of the 20^th^ century); cluster 3 consists of small cities from central and southern regions (most of them with indigenous components); cluster 4 encompasses locations in far regions; and cluster 5 contains mainly small cities from southern regions. This announces a behavior in non-urban zones that approaches to a more modern pattern, a rather common process in social transitions from traditional to modern societies, where some regions adapt faster than others to the institutional innovations [42]. By comparing Tables 3 and 5 as well as Figs 9 and 10, we noticed that cluster 1 from 1920 (53 of 83 departments) is split to form cluster 4, cluster 3, and part of cluster 1 in 1930. These are the departments with less enrollment, but also the three smallest departments in population size. This shows that the predominantly rural character of the country is reduced when compared with 1920. We also notice that in 1930 some departments of cluster 2 behave similar to Concepción. This may allow us to consider that the educational policies between 1920 and 1930, such as compulsory education and institutional changes in the public school system, were also effective for small urban and rural zones, even if they did not eliminate the gap between the outliers and the rest of the departments.

The results obtained with 10-fold cross validation, showed that for 1920, the resulting trees had good predictive performances with high accuracy (0.87 ± 0.09) and a kappa value (0.76 ± 0.14) considered in the higher end of the substantial agreement interval. This showed that for this period, the formed clusters could be well described by the educational attributes. In fact, as shown in Fig 11, out of the 13 educational related attributes (i.e. excluding socio-demographic variables), only four are relevant, being the ‘number of males that do not know how to read’ the most relevant to explain the original differentiation of clusters (root of the tree). The other three attributes are the ‘number of males that know how to read’, the ‘number of women that know how to read’, and the ‘number of *normalist* teachers’. This last attribute is critical to differentiate between the outliers (Santiago, Valparaíso, and Concepción) and the rest of the departments. Each one of the outliers define a unique cluster with a higher proportion of certified teachers than other departments, the so-called *normalist* teachers, i.e. qualified, professional teachers certified by the Normal School of Preceptors (existing in Chile from 1842 to 1974).

The policy that professionalized teaching caused that, in 1920, the number of *normalist* teachers for each 100 enrolled students amounted to 1.8 in Santiago, 1.2 in Valparaíso, 1.9 in Concepción, and 0.7 in the rest of the departments (small-urban and rural zones), while in 1930 these figures grew to 2.1 in Santiago, 1.7 in Valparaíso, 2 in Concepción, 1 in Temuco, and 1 in the rest of the departments. In other words, the professionalization of teaching shows positive results for both the outliers and the other departments. Further, in 1930, the small-urban and rural departments behave as Temuco, the last outlier emerging around the end of the analyzed period.

For 1930, the 10-fold cross validation results showed a decrease in accuracy (0.75 ± 0.18) and in the kappa value (0.66 ± 0.21), although still in the substantial agreement interval but now at the lower end. Also, in comparison to the results of 1920, the dispersion increased. This reflects the difficulty to explain the formed clusters for this period using only the educational related attributes. In fact, we notice that the decision tree shown in Fig 12 is more complex. There are eight attributes, being the ‘number of males in school age (between 6 and 14 years old) that know how to read’ the most relevant attribute (root of the tree). Apart from this attribute (and the same attribute for women), we can appreciate that the ‘total number of schools’, which did not appear in the 1920 decision tree, plays an important role for 1930, distinguishing the behavior of the four outliers (Santiago, Valparaíso, Concepción, and Temuco). Public schools increased in number between 1920 and 1930, but only by a small amount (twenty-five), from 3152 to 3177, while the average enrollment rate of the school-age population increased significantly from 25.10% to 46.11% (*p*-value < 2.2e-16, *d* = 2.03). These figures showed that the school infrastructure had worked at a lower capacity, and that the Law on Compulsory Primary Education showed positive results regarding a more efficient use of resources and infrastructure.

The increase in literacy rates among school-age population reinforces this interpretation. The average illiteracy rate of school-age children fell significantly from 55.28% in 1920 to 24.09% in 1930 (*p*-value < 2.2e-16, *d* = 2.90). Considering literacy as the number of children that know how to read over the total number of school-age children, we notice significant increases in the percentages of literate children for both the big cities and the rest of the departments between 1920 and 1930. In big cities, literacy increased from 60.67% to 82.80% (*p*-value = 0.0002, *d* = 1.67), and from 43.91% to 75.45% in the rest of the departments (*p*-value < 2.2e-16, *d* = 3.08). These results, along with a significant increase in the general attendance to school from 61.42% in 1920 to 70.47% in 1930 (*p*-value = 6.016e-09, *d* = 1.05) can be considered as significant achievements of the policies implemented by the Law on Compulsory Primary Education in the period 1920-1930.

The parliamentary discussions before 1920 criticized the inorganic distribution of public schools, their poor equipment, and their small capacity. As seen, the 1920’s Law on Compulsory Primary Education produced positive effects in all these respects: it professionalized teaching and concentrated students in larger establishments by strategically relocating them in urban and rural centers. In 1927, the Ministry of Education was also created (education affairs were formerly part of the portfolio of the Ministry of Justice), thereby promoting a new centralized management of the educational system. It is not surprising, therefore, that despite the 1929’s world economic crisis, the decade of 1930 continues to show positive results. By analyzing the illiteracy data of the Census of 1940 (which considers the period from 1930 to 1940), we see that the average illiteracy rate decreased significantly from 29.11% in 1930 to 17.92% in 1940 (*p*-value = 2.047e-09, *d* = 1.18); the total number of schools grew from 3,177 in 1930 to 3,599 in 1938 [18]; and the general enrollment increases from 460,950 in 1930 to 524,125 students in 1940 [20]. All these figures confirm that the efforts made in the previous decade were strong enough to institutionalize a new modern education system in Chile, in spite of internal political instabilities (military putsch in 1925 and Ibáñez dictatorship between 1927 and 1931) and external economic pressures (the recession after the 1929’s world crisis).

## Conclusion

Through the application of machine learning to educational data from historical archives, this article addressed the period 1920-1930 of the Chilean history of education in order to assess the consequences of the implementation of the 1920’s Law of Compulsory Primary Education on the Chilean schooling process. We consider the following conclusions.

First, the evaluation of the period 1920-1930 suggests a general positive effect of the 1920’s Law on Compulsory Primary Education. It increased significantly the general attendance, achieved a more efficient use of educational infrastructure, professionalized primary instruction by increasing the number of *normalist* teachers, and improved literacy rates among school-age population in both big cities and small-urban and rural zones. Even though in ten years it could not reverse the trend of concentrating enrollment in urban centers, its policies enhanced the general performance of the schooling process throughout the country.

Second, the dynamics of the schooling process in the period 1920-1930 did not follow a static geospatial configuration. Rather, it produced clusters that experienced changes between 1920 and 1930 regrouping differently and with no correspondence between those departments who are spatially close. Departments with similar attribute values, i.e. belonging to the same cluster, changed their behavior by moving to another group. Nonetheless, while the implemented policies could not remove the gap between big urban centers and the rest of the country in the schooling process, they achieved both a more regular, improved dynamics in small-urban and rural zones and a reinforcement of the schooling process in big urban centers, thereby managing the so-called *crisis of the Republic*.

Third, this work contributed to clarify the transition between a state of social crisis in the Chilean public education (1900-1920) and the aftermath of the crisis (1920-1930). By combining machine learning, historical knowledge, and identifying the specific form of regrouping of the clusters in time and the impact on their attributes, the article shows the potential of transdisciplinary analysis to assess relevant policy measures designed to manage a diagnosed social crisis in historical processes. Thus, this work recovers the dynamic nature of crises by attending to changes in the behavior of the involved variables over time and also offers a new approach to both the history of education in Latin America and the historical methods to reconstruct it.
